# ﻿A multivariate approach to morphological study of shell form in cowries (Gastropoda, Cypraeidae): a case study with *Umbiliaarmeniaca* (Verco, 1912)

**DOI:** 10.3897/zookeys.1158.98868

**Published:** 2023-04-19

**Authors:** Paul C. Southgate, Thane A. Militz

**Affiliations:** 1 School of Science, Technology and Engineering, and Australian Centre for Pacific Islands Research, University of the Sunshine Coast, Maroochydore, Queensland 4556, Australia University of the Sunshine Coast Queensland Australia

**Keywords:** Cowry, gastropod, marine, morphometrics, shell form, taxonomy, *Umbilia*

## Abstract

Multivariate approaches to morphological study of shell form have rarely been applied to cowries (Gastropoda: Cypraeidae) with preference, instead, for comparing formulaic notations of shell form that report averages (i.e., means) for key morphometrics such as shell dimensions, their ratios, and counts of apertural teeth. Although widely applied, the “shell formula” does not account for variation among individuals or support statistical comparison between taxa. This study applied a multivariate approach to analyse shell form within the four accepted subspecies of the cowrie, *Umbiliaarmeniaca* (Verco, 1912) and included a previously unstudied, and most northerly, population of *U.armeniaca* from Lancelin, Western Australia. Multivariate analyses readily separated the recognised subspecies of *U.armeniaca* (*U.a.armeniaca*, *U.a.diprotodon*, *U.a.clarksoni* and *U.a.andreyi*), but did not separate the Lancelin population from *U.a.andreyi*, indicating that the former represents a northward extension of *U.a.andreyi* that is not morphometrically distinguishable. These results provide improved understanding of infraspecific differences in shell form of *U.armeniaca* across its broad distribution, and demonstrate the utility of multivariate morphometric methods for statistical comparison of shell form between taxa. This approach is complimentary to existing research practices and has broad potential application in future morphometric studies of both extant and fossil taxa within the family Cypraeidae.

## ﻿Introduction

The family Cypraeidae Rafinesque, 1815 (cowries) comprises a large group of marine gastropods characterised by colourful, generally glossy, shells with a narrow, elongate aperture bordered by teeth. Cowries demonstrate variable inter- and infraspecific shell morphology (Lorenz 2017a) reflecting evolution, sexual dimorphism, and ecophenotypic plasticity (Schilder and Schilder 1968; Burgess 1970; Tissot 1988; Wilson and Clarkson 2004; Irie 2006; Lorenz and Beals 2013). Where geographically or bathymetrically discrete populations of a species are considered conchologically distinct, reference to a particular population can be aided by assigning subspecies designation. In the most recent review of the family, for example, Lorenz (2017a) recognised 262 living species, of which 42% have at least one subspecies and nearly 20% have more than two subspecies.

Despite broad application of molecular approaches in modern gastropod taxonomy (e.g., Meyer 2003, 2004; Meyer and Paulay 2005), morphological study of shell form remains the primary means of differentiation (Cruz et al. 2012; Lorenz 2017a), and has particular relevance in the study of fossils and of taxa known only from shell remains, for which genetic testing is not possible. Based on precedent established by Vayssière (1910), morphological study of shell form in modern cowrie systematics utilises a formulaic notation, or “shell formula”, to describe various shell traits (i.e., morphometrics) for given populations or taxa. Various arrangements of this notation, reflecting changes to the morphometrics considered, have been employed as a basis for characterising and comparing cowries for more than 100 years (Schilder and Schilder 1938–1939, 1952; Lorenz and Hubert 2000; Lorenz 2001, 2002; Bridges and Lorenz 2013; Lorenz 2017a). While formulaic notations can assist in comparing central tendencies (e.g., mean or median) for a given morphometric between or among *a priori* assigned groups, such as populations or taxa (Bridges and Lorenz 2013), this approach does not account for variation among individuals within groups (Tissot 1984, 1988; Irie 2006) and, according to Landau and Groves (2011), should not be used in isolation to distinguish between taxa. By considering central tendencies in isolation, without accounting for variability, the possibility that putative differences may have arisen by random chance cannot be dismissed, potentially leading to incorrect conclusions. Statistical tests offer a solution to these issues, whereby estimating the probability that differences between central tendencies may have arisen by random chance (i.e., the null hypothesis), a statement of significance can be assigned.

Application of statistical tests to compliment comparisons of shell form between and among groups of cowries can, in its simplest form, assess differences for each morphometric separately (e.g., univariate analysis). When considering a morphometric in this manner (e.g., Lorenz and Beals 2013), only one aspect of morphological variation is represented as independent of other, potentially covariate, morphometrics. It is, therefore, more beneficial to use a multivariate approach to summarise morphological variation. Multivariate approaches, which summarise variation in shell form, are based on multidimensional space where each dimension represents an aspect of morphological variation (a morphometric) and each biological observation (i.e., specimen) can be placed within this space based on their morphometric values. In this manner, morphological configurations of shell form relate mathematically within multidimensional space, based on a measure of resemblance (often distance) between specimens (Mitteroecker and Huttegger 2009). To help comprehend patterns in multidimensional space, ordination techniques can be used to visualise specimens in a new space of reduced dimensions, while maintaining the requirement that similar specimens are closer together than dissimilar ones. Statistical tests can complement visualisation by assessing how a particular morphometric differs among specimens or estimating the probability that *a priori* assigned groups share the same central tendency (i.e., centroid) or variation (i.e., dispersion) within multidimensional space (Guillerme et al. 2020). While prior applications of a multivariate approach for morphological study of cowries have been few (Tissot 1984, 1988), improved availability of open-access software to support multivariate analyses has greatly increased opportunities for their integration into cowrie systematics (e.g., Southgate et al. 2021).

To demonstrate how a multivariate approach to morphological study of shell form might benefit research into cowrie systematics, this study validates existing infraspecific taxonomy for the Australian endemic *Umbiliaarmeniaca* (Verco, 1912). Specific conchological attributes are attributable to location (Wilson and Clarkson 2004) and infraspecific taxonomy has been established by comparing shell formulae and univariate analysis (Lorenz and Beals 2013). Since secondary data for *U.armeniaca* exist in prior studies of shell form (Bridges and Lorenz 2013; Lorenz and Beals 2013), this species provides novel opportunity to demonstrate how primary data from unstudied specimens can supplement secondary data derived from prior studies for both validating existing geographical subspecies and characterising a previously unstudied population of this species.

## ﻿Materials and methods

### ﻿Study populations

**Figure 1.**
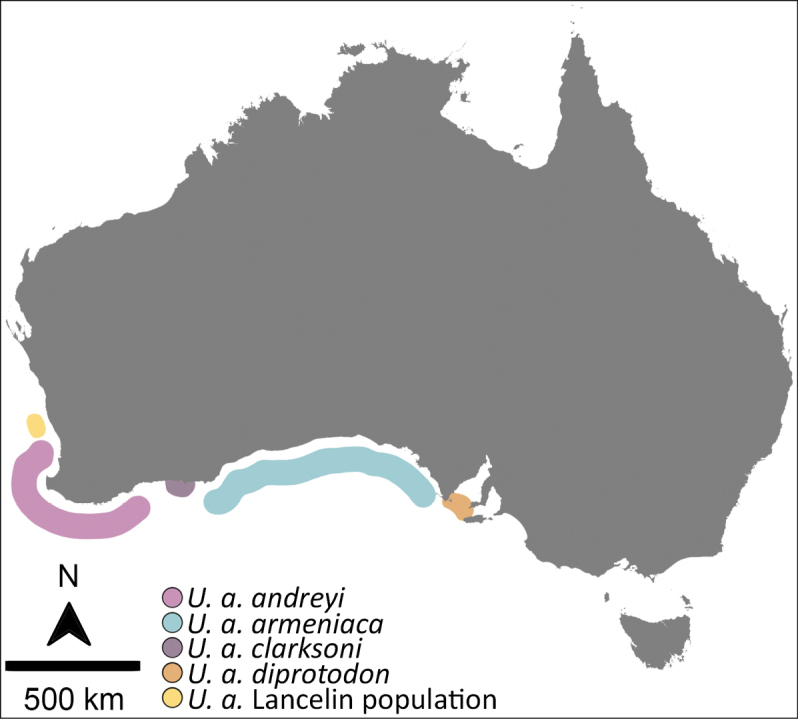
Approximate distributions of the four recognised subspecies of *Umbiliaarmeniaca* and the newly discovered Lancelin population of the species.

The cowrie genus *Umbilia* Jousseaume, 1884 is represented by five living species endemic to Australia. They have limited larval dispersal because of intracapsular development (Wilson 1985) and are characterised by conchologically distinct geographic and bathymetric populations (Wilson and Clarkson 2004). Within the genus, *Umbiliaarmeniaca* has the most extensive geographic range, extending from Kangaroo Island in South Australia to at least Lancelin, north of Perth, in Western Australia, a distance of ~ 3,000 km (Fig. 1). In addition to considerable morphological variation within this range, variation is also apparent within known populations (Bridges and Lorenz 2013; Lorenz and Beals 2013).

**Figure 2.**
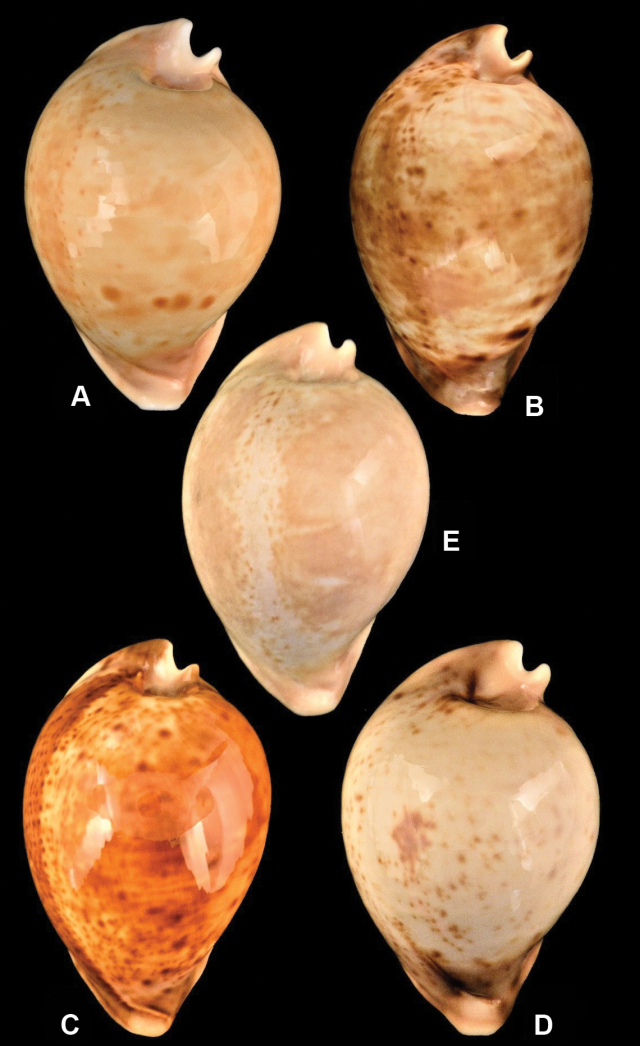
Specimens of the four recognised subspecies of *Umbiliaarmeniaca***A***U.armeniacaarmeniaca*, trawled off Ceduna, Great Australian Bight, 90–120 m, 105 mm **B***U.armeniacadiprotodon*, taken by diver, Thorny Passage, Port Lincoln, South Australia, 35 m, 102 mm **C***U.armeniacaclarksoni*, taken by diver off Cape Le Grande, Esperance, Western Australia, 30–35 m, 94.1 mm **D***U.armeniacaandreyi*, collected using ROV, off Augusta, Western Australia, 150 m, 84.2 mm and **E***U.armeniaca* from the Lancelin population, collected using ROV, off Lancelin, Western Australia, 200 m, 68.9 mm.

Four subspecies of *Umbiliaarmeniaca* are currently recognised (Lorenz and Beals 2013): the nominate *U.a.armeniaca* which ranges across the Great Australian Blight from around the Eyre Peninsula, South Australia, to east of Esperance, Western Australia at depths of ~ 100–200 m (Fig. 2A); *U.a.diprotodon* (Lorenz & Beals, 2013), which ranges from Thorny Passage to western Kangaroo Island and in areas of the Spencer Gulf, South Australia, at depths of 20–60 m (Fig. 2B); *U.a.clarksoni* (Lorenz & Beals, 2013), which is restricted to shallow waters (40–45 m) between Woody Island and Cape Le Grand near Esperance, Western Australia (Fig. 2C); and *U.a.andreyi* (Lorenz & Beals, 2013), which ranges along the south-west coast from Rottnest Island to Windy Harbour, Western Australia, at depths of 100–220 m (Fig. 2D). The four subspecies are considered conchologically distinct, based on comparisons of shell formulae and univariate analysis of selected morphometrics, as well as qualitative differences in colour pattern (Bridges and Lorenz 2013; Lorenz and Beals 2013). In addition to these subspecies, recent exploration using remotely operated vehicles (ROV) discovered a novel population of *U.armeniaca* living at a depth of ~ 200 m off Lancelin, more than 100 km north of what was previously considered the northernmost extent of this species distribution (i.e., Rottnest Island). Only three live specimens have so far been recovered and molecular analysis of this population has not been possible. However, sufficient specimens of complete, empty shells (i.e., collected without tissue) are available to support morphological study of shell form for this northern population (Fig. 2E).

### ﻿Data sources

**Figure 3.**
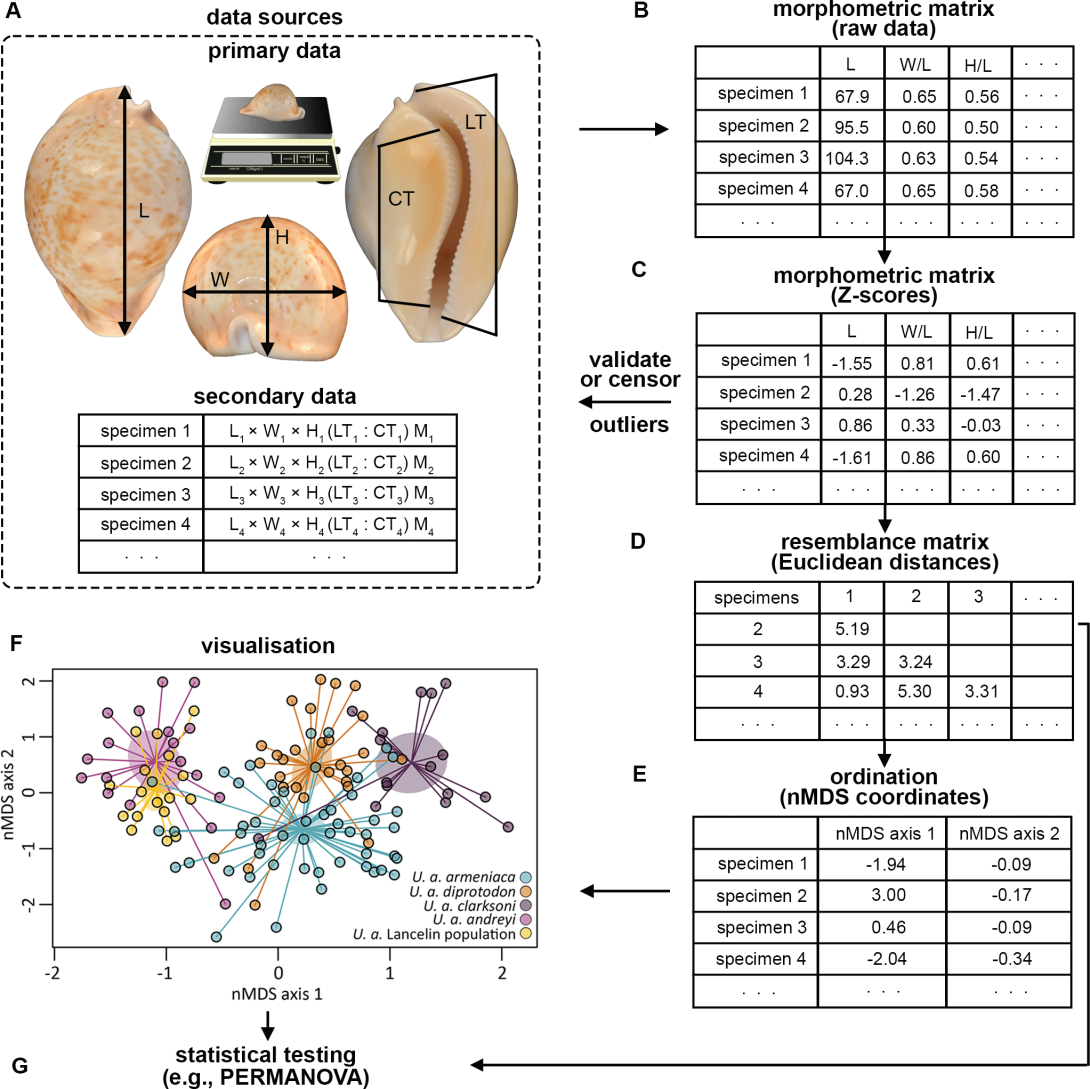
Diagrammatic representation of how a multivariate approach to morphological study of shell form for cowries was applied in this study, using *Umbiliaarmeniaca* as an example (see Suppl. material 1 for an outline of code used) **A** data were sourced by examining unstudied specimens (primary data) or sourced from prior studies (secondary data) **B** data for morphometrics deemed representative of shell form were **C** transformed to *Z*-scores and atypical specimens either validated (primary data) or censored (secondary data) before **D** computing a resemblance matrix based on Euclidean distance between specimens **E** non-metric multidimensional scaling (nMDS) was then used for dimensionality reduction to **F** permit visualisation in a new space of two dimensions and **G** statistical testing was employed to validate visual observations by estimating the probability that a priori assigned groups (taxa or populations) shared the same centroid and dispersion within multidimensional space.

Primary data for shell length (L), shell width (W), and shell height (H), columellar (CT) and labral (LT) tooth counts, and shell mass (M) were collected from previously unstudied specimens of *U.a.armeniaca* (*n* = 21), *U.a.diprotodon* (*n* = 1), *U.a.clarksoni* (*n* = 2) and *U.a.andreyi* (*n* = 4), following the methodology of Lorenz (2017a) (Fig. 3A). Data for the same morphometrics were also collected from previously unstudied specimens of *U.armeniaca* (*n* = 17) originating from the newly discovered population off Lancelin (hereafter “Lancelin population”). Secondary data for L, W, H, CT, LT, and M were sourced from the original descriptions of the *U.armeniaca* subspecies (Lorenz and Beals 2013) and a concurrently published study (Bridges and Lorenz 2013) (Fig. 3A). Only specimens of *U.a.armeniaca* (*n* = 30), *U.a.diprotodon* (*n* = 29), *U.a.clarksoni* (*n* = 15) and *U.a.andreyi* (*n* = 15) with data available for all the above mentioned morphometrics were considered in this study.

### ﻿Data analyses

All data analyses were performed using R (version: 4.2.1), an open-access software environment for statistical testing and graphics, with the *stats* (R Core Team 2022), *vegan* (Oksanen et al. 2022), and *emmeans* (Length 2022) packages. For statistical testing, significance was accepted at a value of *P* < 0.01 to conservatively establish infraspecific differences in shell form. Data summaries are presented in-text as mean (x̄) ± standard deviation (SD).

### ﻿Multivariate methods

The morphometrics considered in this study were those proposed by Bridges and Lorenz (2013) and now commonly adopted for description of Cypraeidae (Lorenz 2017a): shell length (L), height:length ratio (H/L), width:length ratio (W/L), height:width ratio (H/W), normalised columellar tooth count (nCT), normalised labral tooth count (nLT), and relative mass (mR) (Fig. 3B). For each specimen, nCT and nLT were calculated as described by Schilder (1937) and H/L, W/L, H/W and mR were calculated as described by Lorenz (2017a).

Because both dimensionless (e.g., ratios) and differently scaled (e.g., length vs. tooth counts) morphometrics were considered, values were transformed to *Z*-scores prior to ordination and statistical testing (R function: *scale*) (Fig. 3C). Transformation ensured each morphometric was centred, with a mean of zero, and uniformly scaled, with values expressed in terms of deviation from the mean. Morphometric *Z*-scores were then scanned for potential outliers, indicative of atypical specimens. One specimen of *U.a.andreyi* (paratype 13: *Z*_H/L_ = 3.27, *Z*_H/W_ = 6.71), one specimen of *U.a.clarksoni* (paratype 5: *Z*_nLT_ = 3.80), and two specimens of *U.a.diprotodon* (paratype 22: *Z*_W/L_ = 4.23; paratype 33: *Z*_H/W_ = 3.82) were atypical, with at least one morphometric exceeding three standard deviations of the mean (i.e., |*Z*-score| > 3). All were censored from statistical testing since their morphometric values were derived from secondary data that could not be validated (Fig. 3C).

Using the morphometric *Z*-scores for uncensored specimens of *U.a.armeniaca* (*n* = 51), *U.a.diprotodon* (*n* = 28), *U.a.clarksoni* (*n* = 16), *U.a.andreyi* (*n* = 18), and the Lancelin population (*n* = 17), a resemblance matrix was computed based on Euclidean distances between specimens (R function: *vegedist*) (Fig. 3D). Non-metric multidimensional scaling (nMDS) was then used for dimensionality reduction to permit visualisation of the resemblance matrix in a new space of two dimensions (R function: *metaMDS*) (Fig. 3E, F). This ordination technique finds a monotonic relationship between ranks of distances in the resemblance matrix and ranks of distances in a new space of reduced dimensions such that the relative similarity (or dissimilarity) between specimens is represented as closely as possible within the new space. Whilst there are other techniques (e.g., principal component analysis) for ordinating resemblance matrices, nMDS is demonstrably preferred for resolving group differences when studying shell form among Cypraeidae (Tissot 1984).

The influence of each morphometric on the patterns visualised with nMDS (Fig. 3F) was evaluated by testing strength and significance of correlation between each morphometric and the plot configuration (R function: *envfit*). Significant correlations were visualised by fitting a two-dimensional thin-plate generalised additive model spline for the corresponding morphometric as a function of the plot configuration, with results overlayed on the existing nMDS ordination as lines reflecting morphometric clines (R function: *ordisurf*).

Despite numerous advantages of ordination, visual interpretations of multidimensional data after reducing dimensionality can be subjective (Guillerme et al. 2020). Multivariate statistical tests which do not require resemblance matrices to be ordinated are, therefore, useful for objectively validating differences in shell form among groups (Fig. 3G). To test the hypothesis that there were no differences in central tendency of shell form among the five *U.armeniaca* groups examined (i.e., *U.a.armeniaca*, *U.a.diprotodon*, *U.a.clarksoni. U.a.andreyi*, and the Lancelin population) a one-factor permutational analysis of variance (PERMANOVA) was used to fit a linear model to the resemblance matrix (R function: *adonis2*). Pairwise comparisons proceeded detection of a significant group effect, using PERMANOVA for each comparison and controlling for the family-wise error rate with the Holm (1979) procedure. Additionally, to test hypotheses that there were no differences in variation of shell form between any of the *U.armeniaca* groups, permutation-based tests for homogeneity of multivariate dispersions were used to compare the distance of specimens from their group centroid (R function: *permutest.betadisper*), controlling for the family-wise error rate with the Holm (1979) procedure.

### ﻿Univariate methods

To contextualise how the multivariate approach compared with a univariate approach, an analysis of variance (ANOVA), constructed as a linear model, was used to test whether the means of each morphometric differed among the five *U.armeniaca* groups examined (R function: *lm*). Pairwise comparisons between groups were made using linear hypothesis tests of the estimated marginal means (R function: *emmeans*), controlling for the family-wise error rate with the Holm (1979) procedure. Boxplots were used to visualise differences in central tendencies (e.g., mean and median) and variation (e.g., range and quantiles) of morphometrics among groups.

## ﻿Results

### ﻿Multivariate approach

**Figure 4.**
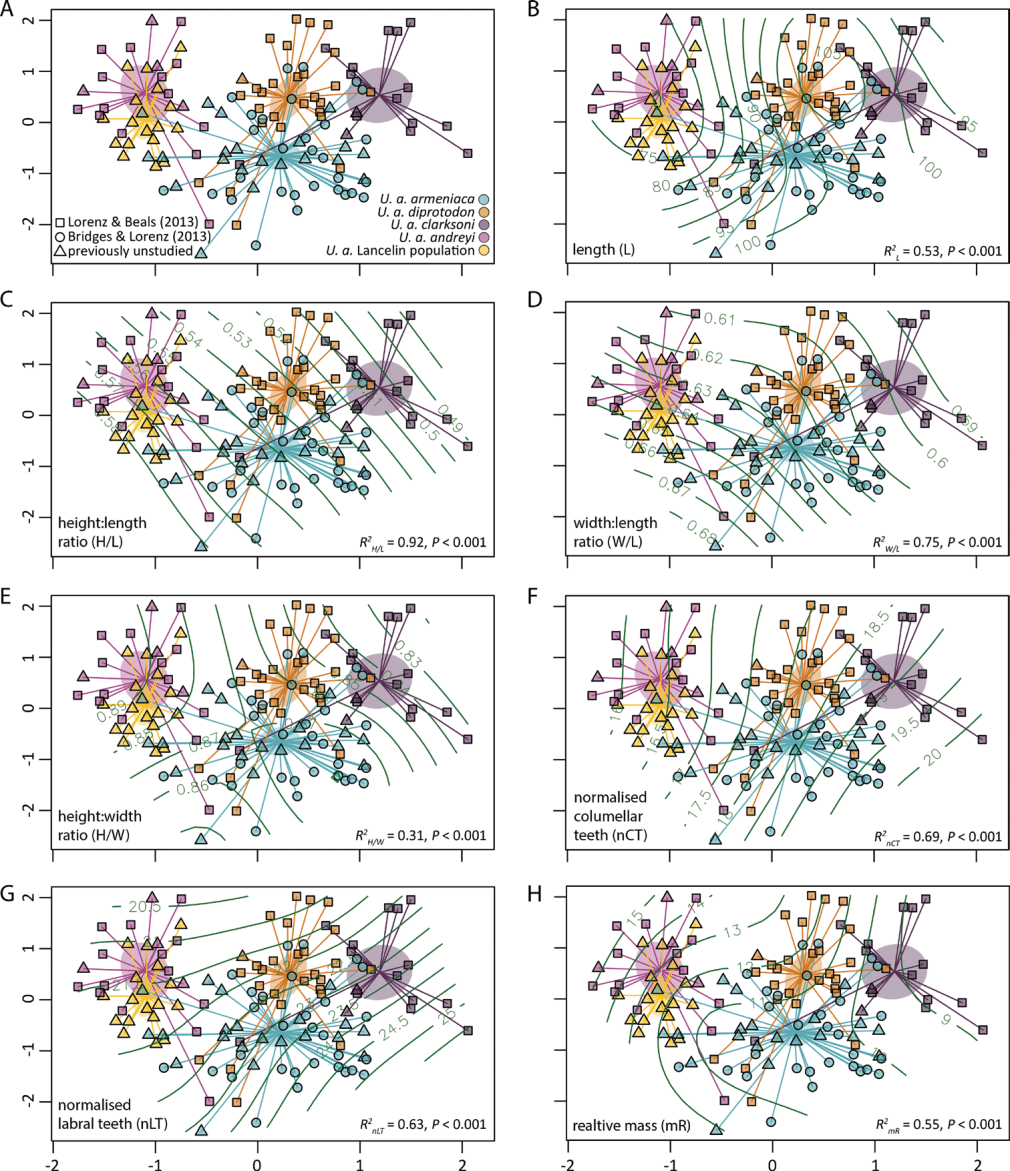
**A**nMDS ordination (stress = 0.16) of the resemblance matrix for *Umbiliaarmeniaca*, where shaded ellipses indicate the 95% confidence interval of group (subspecies or population) centroids and plot characters indicate data source **B–H** Associations between ordination structure and morphometrics influencing this structure, where the green lines illustrate **B** length **C** height:length ratio **D** width:length ratio **E** height:width ratio **F** normalised columellar tooth count **G** normalised labral tooth count, and **H** relative mass contour lines.

Among the studied specimens of *Umbiliaarmeniaca*, the *a priori* assigned groups (i.e., *U.a.armeniaca*, *U.a.diprotodon*, *U.a.clarksoni*, *U.a.andreyi*, and the Lancelin population) were able to explain a significant amount (*R*^2^ = 0.48, *F* = 29.32, *P* < 0.001) of the variation in shell form (Fig. 4A). Between the *U.armeniaca* subspecies, differences in central tendencies (i.e., centroids) of shell form were highly significant (Table 1), with *U.a.andreyi* and *U.a.clarksoni* the most dissimilar (*D* = 4.66, *R^2^* = 0.64, *P* = 0.001) and *U.a.armeniaca* and *U.a.diprotodon* the most similar (*D* = 1.61, *R^2^* = 0.47, *P* = 0.001). Central tendency of shell form for the Lancelin population was distinct from *U.a.armeniaca* (*D* = 2.89, *R^2^* = 0.31, *P* = 0.001), *U.a.clarksoni* (*D* = 4.60, *R^2^* = 0.71, *P* = 0.001), and *U.a.diprotodon* (*D* = 3.41, *R^2^* = 0.56, *P* = 0.001), but was comparable to that of *U.a.andreyi* with the distinction between the two only explaining 9% of the variation in shell form among the corresponding specimens (*D* = 1.00, *R^2^* = 0.09, *P* = 0.012; Fig. 4A, Table 1).

**Table 1.** Results of pairwise comparisons testing the hypotheses that there were no differences in central tendency (i.e., centroid) of shell form among the studied *Umbiliaarmeniaca* groups (subspecies or population). The Euclidean distance (*D*) between centroids, coefficient of determination (*R*^2^), and Holm-adjusted probability that the distance between centroids arose by random chance (*P*) are presented.

*U.armeniaca* group	*andreyi*			*armeniaca*			*clarksoni*			*diprotodon*		
	*D*	*R^2^*	*P*	*D*	*R^2^*	*P*	*D*	*R^2^*	*P*	*D*	*R^2^*	*P*
*armeniaca*	2.86	0.29	0.001	–	–	–	–	–	–	–	–	–
*clarksoni*	4.66	0.64	0.001	2.48	0.23	0.001	–	–	–	–	–	–
*diprotodon*	3.20	0.47	0.001	1.61	0.14	0.001	2.70	0.40	0.001	–	–	–
Lancelin population	1.00	0.09	0.012	2.89	0.31	0.001	4.60	0.71	0.001	3.41	0.56	0.001

Results also indicated that a similar degree of variation (i.e., dispersion) in shell form existed for the *U.armeniaca* subspecies (Fig. 4A, Table 2). For the Lancelin population, variation in shell form was similar to that of *U.a.andreyi* (*t* = 2.96, *P* = 0.041), *U.a.clarksoni* (*t* = 1.66, *P* = 0.568), and *U.a.diprotodon* (*t* = 1.90, *P* = 0.376), but significantly less than that of *U.a.armeniaca* (*t* = 4.80, *P* = 0.001; Fig. 4A, Table 2).

**Table 2.** Results of pairwise comparisons testing the hypotheses that there were no differences in variation (i.e., dispersion) in shell form among the studied *Umbiliaarmeniaca* groups (subspecies or population). The mean (x̄) ± standard deviation (SD) and range in Euclidean distance that specimens were from their group centroid are presented. Shared alphabetic superscripts identify group means that are not significantly (Holm-adjusted *P* ≥ 0.01) different.

*U.armeniaca* group	distance from centroid*	
	(x̄ ± SD)	range
*andreyi*	1.72 ± 0.53^ab^	0.88 – 3.29
*armeniaca*	1.94 ± 0.56^a^	0.87 – 3.24
*clarksoni*	1.53 ± 0.61^ab^	0.73 – 2.91
*diprotodon*	1.50 ± 0.49^ab^	0.63 – 2.59
Lancelin population	1.22 ± 0.46^b^	0.48 – 2.20

All morphometrics considered representative of shell form (i.e., L, H/L, W/L, H/W, nCT, nLT, mR) significantly influenced the ordination structure of the *U.armeniaca* groups visualised in Fig. 4. The most important morphometric (based on *R^2^*) was H/L (*R^2^* = 0.92, *P* < 0.001), followed by W/L (*R^2^* = 0.75, *P* < 0.001), nCT (*R^2^* = 0.69, *P* < 0.001), nLT (*R^2^* = 0.63, *P* < 0.001), mR (*R^2^* = 0.55, *P* < 0.001), L (*R^2^* = 0.53, *P* < 0.001), and H/W (*R^2^* = 0.31, *P* < 0.001). Based on the associations between each morphometric and nMDS plot configuration (Fig. 4B–H), relative differences in shell form could be inferred. For example, shell form of *U.a.andreyi* and the Lancelin population was typified by lesser L and nCT, and greater mR, when compared to the other subspecies. In contrast, shell form of *U.a.clarksoni* was typified by lesser H/L, W/L, H/W and mR, while *U.a.diprotodon* was typified by greater L. *Umbiliaa.armeniaca* was not typified by any extreme in the morphometrics assessed, with a central tendency in shell form intermediate to that of the other subspecies and the Lancelin population.

### ﻿Univariate approach

**Figure 5.**
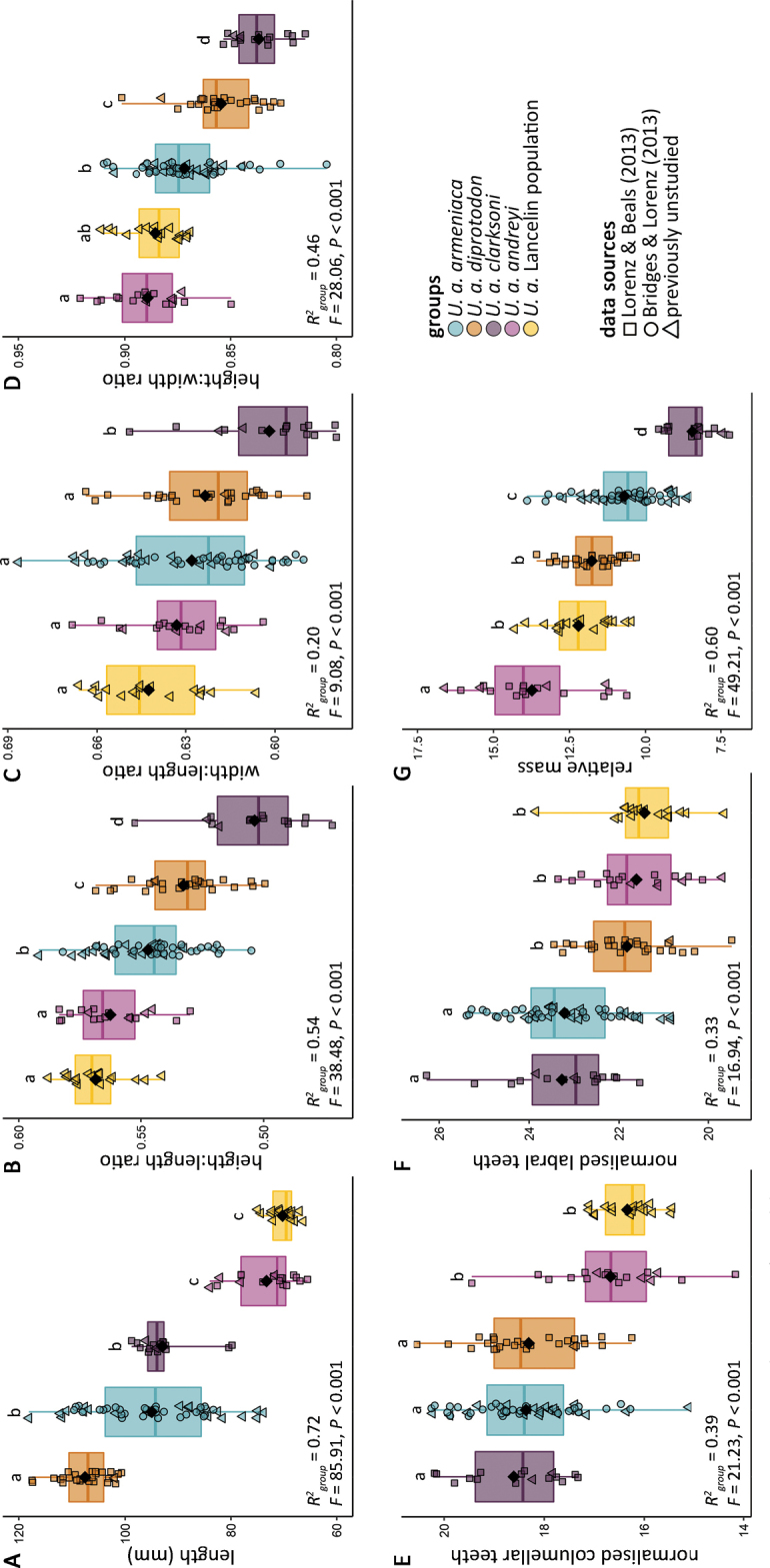
Box plots showing univariate comparisons of **A** shell length **B** height:length ratio **C** width:length ratio **D** height:width ratio **E** normalised columellar tooth count **F** normalised labral tooth count, and **G** relative mass among the studied *Umbiliaarmeniaca* groups (subspecies or population). Black diamonds represent group means, boxes illustrate first and third quartile as box edges and median as central line. Shared alphabetic superscripts identify group means that are not statistically different (Holm-adjusted *P* ≥ 0.01) for a morphometric.

By examining each morphometric independently, relative differences in morphometric values among groups inferred from the nMDS ordination (Fig. 4) could be quantified and validated. For example, the typical shell length of *U.a.diprotodon* was significantly greater than that of any of the other groups considered (Fig. 5A). Likewise, shell form of *U.a.clarksoni* was typified by a significantly lesser H/L, W/L and H/W (Fig. 5B–D). Only three morphometrics (H/L, H/W and mR) differentiated the four subspecies of *U.armeniaca*; shell length was unable to differentiate between *U.a.armeniaca* and *U.a.clarksoni*, W/L was unable to differentiate between *U.a.andreyi*, *U.a.armeniaca*, and *U.a.diprotodon*, nCT was unable to differentiate between *U.a.clarksonii*, *U.a.armeniaca*, and *U.a.diprotodon*, and nLT was unable to differentiate between *U.a.clarksoni* and *U.a.armeniaca* or *U.a.diprotodon* and *U.a.andreyi* (Fig. 5). Measures of central tendency (e.g., mean and median) and variation (e.g., standard deviation and range) for all morphometrics studied are presented separately for each group in Appendix 1.

Considering that not all morphometrics were consistently similar or dissimilar between groups, it was not possible to conclude whether groups differed in overall shell form from univariate comparisons alone. This conundrum is best illustrated by a comparison of *U.a.andreyi* and the Lancelin population, where specimens from these groups differed in central tendencies of relative mass (Fig. 5G). A statistical difference in this morphometric alone, however, did not result in a statistical difference in overall shell form, as determined from the multivariate approach (Fig. 4, Table 1).

## ﻿Discussion

Our results confirm that variation in shell form is prominent within and between populations of *Umbiliaarmeniaca.* Such variability may represent ecophenotypic responses in shell form to environmental factors, random genetic variations independent of adaptive value, or natural selection. Results of this study cannot directly distinguish between these, or other possible causal mechanisms promoting variation in the shell form of cowries, within or between populations, which have seen much discussion elsewhere (see Griffiths 1959; Orr 1959; Kay 1961; Renaud 1976; Tissot 1984, 1988; Irie and Iwasa 2005; Irie 2006). Rather, the following discussion will focus on the implications of such variation, from a systematic perspective, by first discussing the infraspecific taxonomy of *U.armeniaca* and the extent to which our results agree with the putative differences in shell form described by Lorenz and Beals (2013). Consideration of how a multivariate approach to morphological study of shell form might integrate into cowrie systematics is then treated more broadly.

### ﻿Infraspecific taxonomy of *Umbiliaarmeniaca*

Crucial to the validity of taxa differentiated through morphological study of shell form is a replicable and objective approach for comparison. Prior morphological study of shell form in *U.armeniaca* has relied on subjective comparisons of morphometrics and their central tendencies to resolve infraspecific differences, with statistical testing limited to a comparison of relative mass (Bridges and Lorenz 2013; Lorenz and Beals 2013). Given that putative differences were, potentially, an artefact of random chance or subjective inference, the broader application of statistical tests with a clearly defined threshold (*P* < 0.01) for resolving infraspecific differences permits objective validation of the original descriptions of shell form for the subspecies of *U.armeniaca*.

In their original description of *U.armeniaca* subspecies, Lorenz and Beals (2013) qualitatively described the most important infraspecific differences in shell form. The impression that *U.a.clarksoni* is “more elongate and less humped” and has the “lowest dorsal profile” was confirmed in our study by significantly lower H/L, W/L and H/W. Likewise, the impression that *U.a.andreyi* is “slightly more inflated and stunted” and the “most globular and humped” was confirmed by significantly greater H/L and H/W ratios. Significantly lower W/L and H/W of *U.a.diprotodon* relative to *U.a.armeniaca* confirmed the supposition that the former tends to be “less humped” than the latter. The suggestion that *U.a.andreyi* has “fewer teeth on both sides”, however, proved true only if considering raw tooth counts. Once normalised (sensu Schilder 1937), we found the slight difference in nLT between *U.a.andreyi* (21.6 ± 1.0) and *U.a.diprotodon* (21.8 ± 0.9) to be non-significant. Results for our univariate analysis of mR were synonymous with those of Lorenz and Beals (2013), with significant differences between all subspecies. Consideration of these and other morphometric-specific differences, collectively, within our multivariate approach to morphological study of shell form, leads us to conclude that *U.a.armeniaca*, *U.a.diprotodon*, *U.a.clarksoni*, and *U.a.andreyi* are morphologically distinct in central tendencies of shell form. This, however, does not necessarily imply that all specimens of a particular subspecies could be reliably identified based on shell form alone.

Certainly, multivariate distributions (Fig. 4) indicate that while central tendencies of shell form differed significantly, some specimens of a particular subspecies were more representative (i.e., closer to the centroid) of another subspecies. For example, while all specimens of *U.a.diprotodon* were unequivocally distinct from *U.a.andreyi* or *U.a.clarksoni*, some *U.a.diprotodon* paratypes were more representative of *U.a.armeniaca* (paratypes 4, 5, and 11) than *U.a.diprotodon*. Thus, we can conclude that all specimens of *U.a.diprotodon* can be reliably differentiated from *U.a.andreyi* and *U.a.clarksoni*, but not necessarily from *U.a.armeniaca* based on shell form. By the same rationale all specimens of *U.a.clarksoni* could be reliably differentiated from *U.a.andreyi*, but not necessarily from *U.a.diprotodon* or *U.a.armeniaca*, and all specimens of *U.a.andreyi* could be reliably differentiated from *U.a.diprotodon* and *U.a.clarksoni*, but not from *U.a.armeniaca*. None of the subspecies could, therefore, be reliably differentiated from *U.a.armeniaca*, just as *U.a.armeniaca* could not be reliably differentiated from any of the other subspecies. For taxa which cannot be reliably identified from one another based on shell form alone, there is a need to consider additional conchological attributes, beyond the scope of shell form, when assigning specimens of unknown provenance to a particular subspecies.

When comparing taxa, it is important to recognise that not all diagnostic factors can be reliably incorporated into multivariate analysis. Shell pattern, for example, is important in cowrie characterisation (Lorenz 2017a, 2018) and, although quantifiable aspects of shell pattern (e.g., width of dorsal spots) have been included in multivariate analysis of cowries (e.g., Tissot 1984), this is only feasible for taxa with clearly defined patterning. For *U.armeniaca* there exists no common pattern with clearly demarcated boundaries to serve as a basis for replicable and objective quantification (Fig. 2). Rather, Lorenz and Beals (2013) highlighted qualitative differences between the two deep-water subspecies (*U.a.andreyi* and *U.a.armeniaca*), which generally have mottled shells with paler colouration, and the two shallow-water subspecies (*U.a.clarksoni* and *U.a.diprotodon*), which are generally much darker, but with bluish marginal colouration. Other physical features of the shell that may contribute to differentiation between taxa, but are not captured in formulaic or multivariate analyses, include size of the protoconch and prominence of the spire, form of the anterior flange and posterior labral flange, the form of the columellar teeth, dorsal profile, and shape and form of the base, all of which vary among the four subspecies of *U.armeniaca* (Lorenz and Beals 2013). Notwithstanding the inability of multivariate analysis to accommodate all potential conchological attributes of infraspecific variation, the original descriptions of shell form for the *U.armeniaca* subspecies were generally valid when re-evaluated using an objective approach that incorporated statistical tests for resolving infraspecific differences. Considering almost a quarter (24.8%) of the data in our study originated from previously unstudied specimens (*n* = 28) of *U.armeniaca*, the infraspecific differences in shell form outlined by Lorenz and Beals (2013) were found to appropriately generalise the populations of these subspecies.

Of the four *U.armeniaca* subspecies, the nMDS ordination revealed that shells of the previously unstudied Lancelin population were most similar to the neighbouring population of *U.a.andreyi*. Although Lancelin shells had a significantly reduced relative mass, compared to *U.a.andreyi*, this difference was insufficient to differentiate the Lancelin population from *U.a.andreyi* when accounting for the overall variability in shell form. Differences in relative mass are closely associated with differences in shell callosity (Bridges and Lorenz 2013) which is known to correlate with seawater temperature (Tissot 1984; Irie 2006). Other species of cowries (e.g., *Lyncinavitellus*, *Meliceronafelina*, *Monetariaannulus*, *M.caputserpentis*, *Nariaerosa*, *N.helvola*, *N.marginalis*, and *Purpuradustagracilis*) demonstrate latitudinal clines in shell callosity (Schilder and Schilder 1938–1939, 1967; Liversridge 1968; Schilder 1969; Tissot 1984; Irie 2006) and a latitudinal difference in relative mass could therefore be anticipated within the Australian west coast range of *U.armeniaca*. Given a presumed environmental influence on the only morphometric differentiating the Lancelin population from *U.a.andreyi*, and overall similarities in shell form, we conclude that the Lancelin population of *U.armeniaca* is best considered a northern extension of the distribution of *U.a.andreyi*.

### ﻿Considerations for a multivariate approach to cowrie systematics

Most datasets used in the study of shell form are multidimensional and, consequently, a large component of any systematic study will involve consideration of how to extract a meaningful summary of differences in shell form among specimens and/or groups. The multivariate approach taken in this study, couples easy-to-interpret graphics produced via ordination with an objective appraisal of inter-group differences via statistical testing. When combined with a univariate approach, as done here, a broad range of questions of relevance to systematics can be addressed objectively. For example, if the goal is to characterise differences in shell form between groups, multivariate tests comparing differences in central tendencies (such as group centroids) or variability (such as group dispersion) in multidimensional space are most appropriate. Furthermore, if the goal is to characterise how a particular morphometric differs between groups, univariate tests comparing differences in central tendencies (such as group means) for that morphometric will be of value. Regardless of the tests used, statistical testing should only be employed to address questions framed within an appropriate statistical context if sample sizes are large enough to permit detection of statistically significant differences (Cohen 1988; Anderson 2001).

It is also important to consider which morphometrics are most appropriate for statistical testing. Studies comparing shell form among cowries have, over time, varied greatly in the morphometrics selected to represent shell form (Bridges and Lorenz 2013). While theoretically there is no limit to the number of morphometrics which can be incorporated into multivariate analyses, the “curse of dimensionality” (Bellman 1966) favours minimising the number selected from a theoretical perspective. Additionally, for each unique measurement there exists potential for observational error and, where morphometrics make assumptions concerning growth (e.g., Irie 2006), modelling error. Furthermore, some morphometrics (e.g., whorl widths or callus thickness) require cross-sectioning of shells (Tissot 1988; Irie 2006) which is not feasible in situations where destructive sampling is not possible, such as when working with type specimens or those held in public collections. Other morphometrics are inherently taxon-specific (e.g., colouration and/or patterning) which complicates comparisons with taxa lacking the associated trait (Guillerme et al. 2020). Finally, many morphometrics have isometric or allometric relationships and are positively correlated with one another, such that they represent similar sources of variability in shell form (Tissot 1984, 1988). Taken together, these factors incentivise limiting selection to shared morphometrics that are sampled non-destructively and represent unique sources of variation in shell form among cowries. From past studies most of the meaningful variation in shell form among cowries is known to come from shell size, shape, callosity, and the number of basal teeth (Tissot 1984; 1988). For example, when considering 18 morphometrics as part of a multivariate approach to study shell form in *Monetariacaputserpentis*, Tissot (1984) found that three components of variation (shell size, shape and callosity, and number of basal teeth) accounted for almost 70% of the total variation in shell form among populations sampled across the geographical range of this species. On this basis, care was taken to ensure that the seven morphometrics selected to represent shell form in the present study uniquely represented variation in shell size (i.e., length), shape (i.e., height:length, width:length, and height:width ratios), callosity (i.e., relative mass), and number of basal teeth (i.e., normalised columellar and labral tooth counts).

Aside from representing the primary sources of infra- and interspecific variation in shell form of cowries (Tissot 1984, 1988), a further advantage of these seven morphometrics is that they require only four shell measurements taken with a vernier calliper (e.g., length, width, height) or balance (e.g., mass) and counts of basal teeth, all of which are sampled non-destructively. For similar reasons, the morphometrics considered in our study were also proposed by Bridges and Lorenz (2013), and are commonly adopted (e.g., Lorenz 2017b) for description of Cypraeidae. While acknowledging that morphological studies with other gastropod families have developed methods for quantifying the variation in shell shape from a set of homologous points, or landmarks, positioned on images using the Procrustes method (e.g., Rohlf and Marcus 1993), such methods have not been developed for the Cypraeidae. Gastropods such as Muricidae, for which such methods have been developed, have an exposed shell spire and numerous ribs and spines which provides a broad range of appropriate landmarks for such methods (Doyle et al. 2018; Bocxlaer et al. 2020; Larsson et al. 2020). This is not the case for cowries, however, which demonstrate determinate growth (Vermeij and Signor 1992), generally have a concealed spire, and lack meristic shell ornamentations, such as spines and varices. Furthermore, such methods were developed with the primary intention of detecting minute variation within populations related to ontogenetic development patterns (Larsson et al. 2020) or environmental selective pressures (Doyle et al. 2018; Bocxlaer et al. 2020) rather than establishing novel taxonomic characters. Given the intent of infraspecific taxonomy of cowries to distinguish between populations that are visibly distinct (Lorenz 2017a), an approach that considers morphometrics which are easy to comprehend, such as those used in this study, would seem most appropriate.

## ﻿Conclusions

Multivariate approaches to the morphological study of shell form of cowries have been utilised primarily to develop hypotheses related to ecological and functional diversity within the target species (Tissot 1984, 1988). Although used more recently in recognition and description of a new species of cowrie from the fossil record (Southgate et al. 2021), integration of a multivariate approach into cowrie systematics has received surprisingly little attention, with univariate comparison of central tendencies, using shell formulae, predominant in recent comparative studies (Bridges and Lorenz 2013; Lorenz 2017a) and in recognising new taxa (e.g., Lorenz 2017b). While useful in describing key morphometric characters and allowing subjective comparisons between taxa (Bridges and Lorenz 2013), including fossils (Landau and Groves 2011; Southgate and Roberts 2022), the shell formula, as applied to cowries, does not convey variability of key morphometric characters, nor does it support the testing of statistical differences between populations or taxa.

This study has demonstrated the utility of a multivariate approach that couples easy-to-interpret graphics produced via ordination with an objective appraisal of inter-group differences via statistical testing with clearly defined thresholds for both outlier detection (i.e., |*Z*-score| > 3) and resolving infraspecific differences (*P* < 0.01). Using *Umbiliaarmeniaca* as a case study, we showed how primary data from unstudied specimens might supplement secondary data from prior studies to validate existing infraspecific taxonomy and characterise a previously unstudied (Lancelin) population of this species. The multivariate approach showed the four recognised *U.armeniaca* subspecies to be similarly variable, but confirmed differences in central tendency of shell form. Our analysis did not justify differentiation of the Lancelin population of *U.armeniaca* which is best considered a northward extension of *U.a.andreyi*. Results of this study provide improved understanding of intraspecific differences in shell form of *U.armeniaca* across its broad distribution, and demonstrate how multivariate morphometric methods for statistical comparison of shell form between taxa might benefit cowrie systematics. This approach is complimentary to existing research practices and has broad potential application in future morphometric based studies of cowries.

## ﻿Appendix 1

**Table A1.** Measures of central tendency (i.e., mean and median) and variation (i.e., standard deviation and range) among the studied *Umbiliaarmeniaca* groups (subspecies or population) for each morphometric considered representative of shell form. Shared alphabetic supercrips identify group means that are not statistically different (Holm-adjusted *P* ≥ 0.01) for a morphometric.

Morphometric	*Umbiliaarmeniaca* groups				
	*andreyi*	*armeniaca*	*clarksoni*	*diprotodon*	Lancelin
**length (mm)**					
x̄ ± SD	73.3 ± 5.8^c^	94.9 ± 11.1^b^	92.9 ± 5.4^b^	107.5 ± 4.5^a^	70.2 ± 2.5^c^
median	71.3	94.2	93.9	107.0	69.5
range	65.5–84.0	73.9–118.1	79.8–98.8	100.7–117.5	66.3–75.0
**height:length ratio**					
x̄ ± SD	0.563 ± 0.016^a^	0.547 ± 0.018^b^	0.504 ± 0.020^d^	0.533 ± 0.017^c^	0.569 ± 0.013^a^
median	0.566	0.545	0.502	0.531	0.570
range	0.530–0.584	0.505–0.592	0.472–0.553	0.500–0.569	0.541–0.588
**width:length ratio**					
x̄ ± SD	0.633 ± 0.017^ab^	0.628 ± 0.023^ab^	0.602 ± 0.019^c^	0.623 ± 0.019^b^	0.642 ± 0.017^a^
median	0.632	0.622	0.596	0.619	0.646
range	0.604–0.668	0.590–0.687	0.579–0.649	0.589–0.664	0.606–0.666
**height:width ratio**					
x̄ ± SD	0.889 ± 0.017^a^	0.872 ± 0.020^b^	0.837 ± 0.012^d^	0.854 ± 0.017^c^	0.885 ± 0.014^a^
median	0.889	0.874	0.838	0.857	0.884
range	0.850–0.921	0.805–0.853	0.815–0.853	0.826–0.901	0.869–0.911
**normalised columellar teeth**					
x̄ ± SD	16.7 ± 1.2^b^	18.4 ± 1.1^a^	18.6 ± 1.0^a^	18.3 ± 1.0^a^	16.3 ± 0.5^b^
median	16.7	18.4	18.4	18.5	16.2
range	14.2–19.4	15.1–20.3	17.3–20.2	16.2–20.5	15.4–17.1
**normalised labral teeth**					
x̄ ± SD	21.6 ± 1.0^b^	23.2 ± 1.2^a^	23.3 ± 1.3^a^	21.8 ± 0.9^b^	21.4 ± 0.9^b^
median	21.8	23.4	23.0	21.9	21.6
range	19.7–23.4	20.8–25.4	21.5–26.3	19.5–23.4	19.6–23.9
**relative mass**					
x̄ ± SD	13.7 ± 1.7^a^	10.7 ± 1.2^c^	8.5 ± 0.7^d^	11.8 ± 0.9^b^	12.2 ± 1.1^b^
median	14.0	10.6	8.3	11.8	12.2
range	10.6–16.6	8.6–13.9	7.2–9.6	10.3–13.6	10.5–14.3

## Supplementary materials

Supplementary material 1 Annotated code pertaining to the multivariate approach Data type docx file https://binary.pensoft.net/file/840031This dataset is made available under the Open Database License (http://opendatacommons.org/licenses/odbl/1.0/). The Open Database License (ODbL) is a license agreement intended to allow users to freely share, modify, and use this Dataset while maintaining this same freedom for others, provided that the original source and author(s) are credited.Paul C. Southgate, Thane A. Militz
