# Supplementary material for: ﻿A multivariate approach to morphological study of shell form in cowries (Gastropoda, Cypraeidae): a case study with Umbiliaarmeniaca (Verco, 1912)
Source: Zookeys. 2023 Apr 19;1158:69–89. doi: 10.3897/zookeys.1158.98868 (PMC10193853; doi:10.3897/zookeys.1158.98868)
Supplement: Supplementary material 1 — Annotated code pertaining to the multivariate approach [file zookeys-1158-069_article-98868__-s001.docx]

Supplementary Material

# Annotated code pertaining to the multivariate approach presented in:
 # Southgate, P.C., Militz, T.A. 2023 A multivariate approach to morphological study of shell form in cowries (Gastropoda, Cypraeidae): a case study with Umbilia armeniaca (Verco, 1912).
 # Abbreviations
 # H, height (mm)
 # W, width (mm)
 # L, length (mm)
 # H.L, height to length ratio
 # W.L, width to length ratio
 # H.W, height to width ratio
 # LT, labral teeth (count)
 # nLT, normalized labral teeth (count) - based on Schilder (1937)
 # CT, columellar teeth (count)
 # nCT, normalized columellar teeth (count) - based on Schilder (1937)
 # M, mass (g)
 # mR, relative mass - based on Bridges and Lorenz (2012)

# Packages required
library(vegan) # for vegdist, metaMDS, ordiellipse, ordispider, envfit, ordisurf, adonis2 (PERMANOVA)
library(pairwiseAdonis) # wrapper function for pairwise comparisons using adonis2 (Permanova) from package 'vegan' (available: https://github.com/pmartinezarbizu/pairwiseAdonis)

# Import data
dat <- read.csv("Umbilia_armeniaca.csv") # import data (morphometrics and grouping factors as columns; studied specimens as rows)
dat$group <- as.factor(dat$group) # designate a priori groups as factors (subspecies/population)

# Calculate morphometrics
dat$H.L <- dat$H/dat$L # height to length ratio
dat$H.W <- dat$H/dat$W # height to width ratio
dat$W.L <- dat$W/dat$L # width to length ratio
dat$nLT <- 7 + (dat$LT - 7) * sqrt(25/dat$L) # normalised labral teeth
dat$nCT <- 7 + (dat$CT - 7) * sqrt(25/dat$L) # normalised columellar teeth
dat$mR <- dat$M/(dat$L * dat$W * dat$H * 0.00293)*100 # relative mass

# Create morphometric matrix
morph <- dat[,c("L","H.L","H.W","W.L","nLT","nCT","mR")] # specify morphometrics deemed representative of shell form

# Transform data to Z-scores and identify atypical specimens
morph <- as.data.frame(lapply(morph, scale)) # transform data for each morphometric to Z-scores
which(abs(morph) > 3, arr.ind = TRUE)[,1] # identify atypical specimens (-3 < Z-score < 3) by row number

# Omit atypical specimens from further analyses (optional - recommended for secondary data when validation is not possible)
dat <- dat[-c(which(abs(morph) > 3, arr.ind = TRUE)[,1]),]
morph <- morph[-c(which(abs(morph) > 3, arr.ind = TRUE)[,1]),]

# Create resemblance matrix
dist <- vegdist(morph, method = "euclidean") #resemblance matrix of similarity, based on Euclidean distances, between specimens

# Ordinate resemblance matrix in reduced dimensional space
morphMDS <- metaMDS(dist, k =2, try = 100, trymax =100, stress = 1, autotransform = FALSE) #nMDS based on two dimensions
morphMDS #examine stress (as a general rule: 0 = perfect fit, < 0.15 ideal fit, < 0.20 good fit, < 0.25 okay fit, >/= 0.25 uninformative fit)

# Visualise nMDS ordination
nMDS_plot <- function(x) {
 plot(x, type = "n")
 ordiellipse(x, groups = dat$group, col = unique(as.numeric(dat$group)), kind = "se", conf = 0.95, draw = "polygon", lty = 0, alpha = 0.40) # 95% confidence ellipse for group centroids.
 ordispider(x, groups = dat$group, col = unique(as.numeric(dat$group))) # draws lines connecting specimens to group centroid
 points(x, pch = 21, bg = unique(as.numeric(dat$group))[dat$group]) # specimens as point
 legend('topright', title = "group", legend = levels(dat$group), col = unique(as.numeric(dat$group)), pch = 16, cex = 0.6, y.intersp = 0.7, bty = "n")
}
nMDS_plot(morphMDS) # nMDS ordination to plot

# Strength and significance of correlation between each morphometric and nMDS ordination
envfit(morphMDS ~ group + L + W.L + H.L + H.W + nCT + nLT + mR, data = dat, perm = 9999) #r2 (strength) and Pr (significance) of each morphometric

ordisurf(morphMDS, dat$L, add = TRUE, col = "darkgreen", labcex = 0.8) # add specified morphometric to nMDS plot

# Test for differences in central tendency (i.e. centroid) of shell form among groups
adonis2(dist ~ group, perm = 9999, data = dat) # one-factor permutational analysis of variance (PERMANOVA) to test if there were differences in shell form among groups
pairwise.adonis(dist, factors = dat$group, perm = 9999, p.adjust.m = "holm") # pairwise comparisons, using PERMANOVA for each comparison and controlling for family-wise error rate with Holm (1979) procedure

# Test for differences in variation (i.e. dispersion) of shell form between groups
dispersion <- betadisper(dist, type = c("centroid"), group = dat$group) # calculates group dispersion
tapply(dispersion$distances, dat$group, mean) # average dispersion for each group
tapply(dispersion$distances, dat$group, sd) # standard deviation of dispersion for each group
dispersion.test <- permutest(dispersion, pairwise = TRUE, permutations = 9999) #permutation-based test of differences in dispersion between and among groups
p.adjust(dispersion.test$pairwise$permuted, method = c("holm")) # permuted p-values, controlling for family-wise error rate with Holm (1979) procedure

#END
